# *N*′-(4-Bromo­phenyl­sulfon­yl)isonicotino­hydrazide

**DOI:** 10.1107/S1600536809028475

**Published:** 2009-07-25

**Authors:** Islam Ullah Khan, Muhammad Ashfaq, Muhammad Nadeem Arshad, Hamad Ahmad, Ghulam Mustafa

**Affiliations:** a Materials Chemistry Laboratory, Department of Chemistry, GC University, Lahore, Pakistan; b Department of Chemistry, University of Gujrat, Gujrat, Pakistan

## Abstract

The title compound, C_12_H_10_BrN_3_O_3_S, crystallizes with two crystallographically independent mol­ecules in the asymmetric unit. The dihedral angles between the two six-membered rings in the mol­ecules are 34.1 (3) and 45.1 (2)°. In the crystal structure, mol­ecules are connected *via* N—H⋯O and N—H⋯N hydrogen bonding.

## Related literature

For general background to isonicotinic acid hydrazides, see: Carlton (1967 ▶). For a related structure, see: Wang *et al.* (2008 ▶). For the synthesis and biological activity of isoniazid and hydrazide derivatives, see: Lourenco *et al.* (2008 ▶); Kucukguzel *et al.* (2003 ▶); Carvalho *et al.* (2008 ▶), For graph-set notation, see: Bernstein *et al.* (1995 ▶).
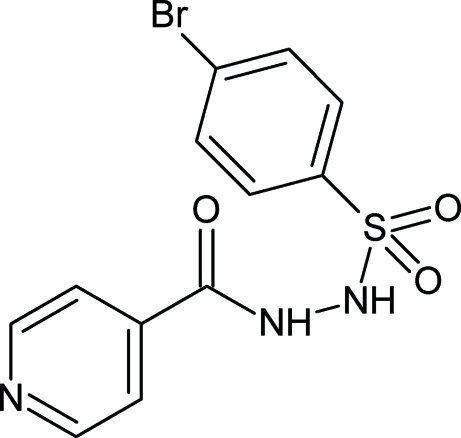


## Experimental

### 

#### Crystal data

C_12_H_10_BrN_3_O_3_S
*M* *_r_* = 356.20
Monoclinic, 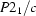

*a* = 10.1229 (6) Å
*b* = 19.0440 (12) Å
*c* = 15.0640 (7) Å
β = 96.862 (2)°
*V* = 2883.2 (3) Å^3^
*Z* = 8
Mo *K*α radiation
μ = 3.01 mm^−1^
*T* = 296 K
0.36 × 0.30 × 0.15 mm

#### Data collection

Bruker Kappa APEXII CCD diffractometer
Absorption correction: multi-scan (*SADABS*; Bruker, 2007 ▶) *T*_min_ = 0.349, *T*_max_ = 0.641
29398 measured reflections
6601 independent reflections
3473 reflections with *I* > 2σ(*I*)
*R*_int_ = 0.053

#### Refinement

*R*[*F* ^2^ > 2σ(*F* ^2^)] = 0.053
*wR*(*F* ^2^) = 0.146
*S* = 1.01
6601 reflections
373 parameters
H atoms treated by a mixture of independent and constrained refinement
Δρ_max_ = 1.06 e Å^−3^
Δρ_min_ = −0.88 e Å^−3^

### 

Data collection: *APEX2* (Bruker, 2007 ▶); cell refinement: *SAINT* (Bruker, 2007 ▶); data reduction: *SAINT*; program(s) used to solve structure: *SHELXS97* (Sheldrick, 2008 ▶); program(s) used to refine structure: *SHELXL97* (Sheldrick, 2008 ▶); molecular graphics: *ORTEP-3 for Windows* (Farrugia, 1997 ▶) and *PLATON* (Spek, 2009 ▶); software used to prepare material for publication: *WinGX* (Farrugia, 1999 ▶) and *PLATON*.

## Supplementary Material

Crystal structure: contains datablocks I, global. DOI: 10.1107/S1600536809028475/nc2151sup1.cif

Structure factors: contains datablocks I. DOI: 10.1107/S1600536809028475/nc2151Isup2.hkl

Additional supplementary materials:  crystallographic information; 3D view; checkCIF report

## Figures and Tables

**Table 1:** Hydrogen-bond geometry (Å, °)

*D*—H⋯*A*	*D*—H	H⋯*A*	*D*⋯*A*	*D*—H⋯*A*
N2—H2⋯O4^i^	0.76 (4)	2.15 (4)	2.882 (4)	165.09
N3—H3⋯N4^ii^	0.78 (4)	2.10 (4)	2.868 (5)	168.21
N6—H6⋯O6^iii^	0.83 (4)	2.26 (4)	2.998 (4)	147.35
N5—H5⋯N1^iv^	0.83 (4)	2.03 (4)	2.847 (4)	168.32
N3—H3⋯O1	0.78 (4)	2.49 (4)	2.732 (4)	100 (3)

Symmetry codes: (i) 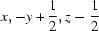; (ii) 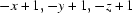; (iii) 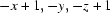; (iv) 
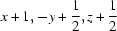
.

**Fig. 1.**
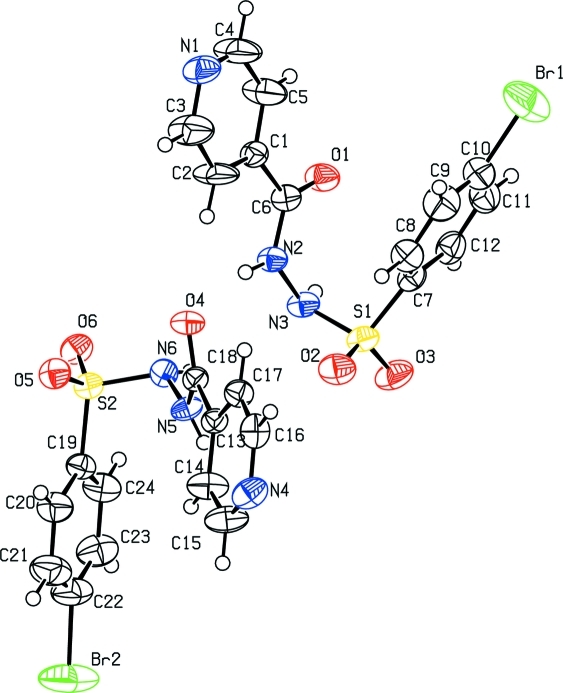
The structure of (I) with labeling and displacement ellipsoids drawn at the 50% probability level.

**Fig. 2.**
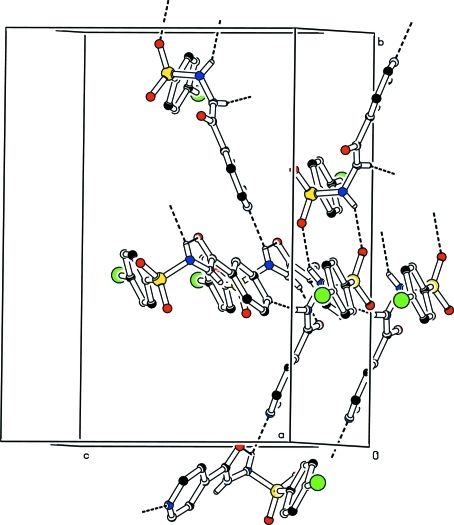
Unit cell packing for (I) with hydrogen bonding shown as dashed lines.

## supplementary crystallographic information

### Comment

Isonicotinic acid hydrazide(INH) commonly known as isoniazid is a drug being used for the treatment of tuberclosis (TB) for long time (Carlton, 1967). Different approaches have been made for the synthesis of biologically active derivatives of isoniazid (Lourenco *et al.*, 2008), (Kucukguzel *et al.*, 2007), (Carvalho, *et al.*. 2008) and their crystallographic studies (Wang *et al.*, 2008). In this context we report the crystal structure of title compound (*N*'-[(4-bromophenyl)sulfonyl]pyridine-4-carbohydrazide) a sulfonamide derivative of Isoniazid.

The title compound crystallizes with two crystallographically independent molecules in the asymmetric unit. The dihedral angle in each of these molecules amount to 34.1 (3) ° in molecule A, while it is 45.1 (2) ° (Fig. 1).

In the crystal structure the molecules are connected via intermolecular and intramolecular N–H···O and N–H···N hydrogen bonding (Fig. 2 and Tab. 1). One of the two indpenendent molecules is connected into dimers via N–H···O hydrogen bonding of the sulfonamide group into *R*_2_^2^(8) rings (Bernstein *et al.*, 1995). These dimers are further linked  by additional N–H···O and N–H···N hydrogen bonding.

### Experimental

To the solution of Isoniazid (0.5 g, 3.646 mmol) in distilled water (10 ml), 4-Bromobenzenesulfonyl chloride(0.9316 g, 3.65 mmol) was suspended. The reaction mixture was stirred at room temperature for 4 hrs at constant pH 8–9, which was adjusted by 1*M* sodium carbonate solution. After completion of the reaction which was observed by the consumption of suspended 4-Bromobenzenesulfonyl chloride, the pH was adjusted at 2–3 using 1 N HCl solution, which results the formation of a light yellow coloured precipitate, which was filtered off and dried. The prodcut was recrystallized from methanol.

### Refinement

The C-H H-atoms were positioned with idealized geometry with C—H = 0.93 Å and were refined using a riding model with *U*_iso_(H) = 1.2 *U*_eq_(C). The N-H H atoms were located in difference map and refined isotropic ( *U*_iso_(H) = 1.2 *U*_eq_(N) with varying coordinates.

### Crystal data

**Table:**

C_12_H_10_BrN_3_O_3_S	*F*(000) = 1424
*M**_r_* = 356.20	*D*_x_ = 1.641 Mg m^−^^3^
Monoclinic, *P*2_1_/*c*	Mo *K*α radiation, λ = 0.71073 Å
Hall symbol: -P 2ybc	Cell parameters from 5184 reflections
*a* = 10.1229 (6) Å	θ = 2.1–22.9°
*b* = 19.0440 (12) Å	µ = 3.01 mm^−^^1^
*c* = 15.0640 (7) Å	*T* = 296 K
β = 96.862 (2)°	Needle, white yellow
*V* = 2883.2 (3) Å^3^	0.36 × 0.30 × 0.15 mm
*Z* = 8	

### Data collection

**Table:**

Bruker Kappa APEXII CCD diffractometer	6601 independent reflections
Radiation source: fine-focus sealed tube	3473 reflections with *I* > 2σ(*I*)
graphite	*R*_int_ = 0.053
φ and ω scans	θ_max_ = 27.5°, θ_min_ = 2.1°
Absorption correction: multi-scan (*SADABS*; Bruker, 2007)	*h* = −13→11
*T*_min_ = 0.349, *T*_max_ = 0.641	*k* = −24→23
29398 measured reflections	*l* = −17→19

### Refinement

**Table:**

Refinement on *F*^2^	Primary atom site location: structure-invariant direct methods
Least-squares matrix: full	Secondary atom site location: difference Fourier map
*R*[*F*^2^ > 2σ(*F*^2^)] = 0.053	Hydrogen site location: inferred from neighbouring sites
*wR*(*F*^2^) = 0.146	H atoms treated by a mixture of independent and constrained refinement
*S* = 1.01	*w* = 1/[σ^2^(*F*_o_^2^) + (0.0597*P*)^2^ + 2.5287*P*] where *P* = (*F*_o_^2^ + 2*F*_c_^2^)/3
6601 reflections	(Δ/σ)_max_ = 0.001
373 parameters	Δρ_max_ = 1.06 e Å^−^^3^
0 restraints	Δρ_min_ = −0.88 e Å^−^^3^

### Special details

**Table:**

Geometry. All esds (except the esd in the dihedral angle between two l.s. planes) are estimated using the full covariance matrix. The cell esds are taken into account individually in the estimation of esds in distances, angles and torsion angles; correlations between esds in cell parameters are only used when they are defined by crystal symmetry. An approximate (isotropic) treatment of cell esds is used for estimating esds involving l.s. planes.
Refinement. Refinement of F^2^ against ALL reflections. The weighted R-factor wR and goodness of fit S are based on F^2^, conventional R-factors R are based on F, with F set to zero for negative F^2^. The threshold expression of F^2^ > 2sigma(F^2^) is used only for calculating R-factors(gt) etc. and is not relevant to the choice of reflections for refinement. R-factors based on F^2^ are statistically about twice as large as those based on F, and R- factors based on ALL data will be even larger.

### Fractional atomic coordinates and isotropic or equivalent isotropic displacement parameters (Å^2^)

**Table:**

	*x*	*y*	*z*	*U*_iso_*/*U*_eq_	
Br1	0.01531 (7)	0.40221 (5)	0.59324 (5)	0.1083 (3)	
Br2	1.07622 (6)	0.14607 (4)	0.37352 (6)	0.1046 (3)	
S1	0.46424 (10)	0.38694 (6)	0.33172 (7)	0.0392 (3)	
S2	0.46755 (9)	0.10499 (5)	0.43498 (6)	0.0328 (2)	
O1	0.1616 (3)	0.49151 (15)	0.28196 (19)	0.0467 (7)	
O2	0.4756 (3)	0.31692 (15)	0.2999 (2)	0.0510 (8)	
O3	0.5802 (3)	0.42527 (17)	0.3664 (2)	0.0566 (9)	
O4	0.2996 (2)	0.20910 (13)	0.59686 (17)	0.0376 (7)	
O5	0.3909 (3)	0.16199 (14)	0.39574 (18)	0.0420 (7)	
O6	0.4282 (3)	0.03415 (14)	0.41370 (17)	0.0413 (7)	
N1	−0.2166 (3)	0.3447 (2)	0.1433 (3)	0.0533 (10)	
N2	0.2699 (3)	0.41007 (18)	0.2107 (2)	0.0340 (8)	
H2	0.270 (4)	0.383 (2)	0.174 (3)	0.041*	
N3	0.3969 (3)	0.43457 (18)	0.2456 (2)	0.0353 (8)	
H3	0.398 (4)	0.474 (2)	0.259 (3)	0.042*	
N4	0.5582 (4)	0.42464 (18)	0.6902 (2)	0.0474 (9)	
N5	0.5095 (3)	0.17527 (17)	0.5802 (2)	0.0327 (8)	
H5	0.590 (4)	0.176 (2)	0.600 (3)	0.039*	
N6	0.4645 (3)	0.11035 (17)	0.5443 (2)	0.0336 (8)	
H6	0.493 (4)	0.077 (2)	0.577 (3)	0.040*	
C1	0.0288 (4)	0.4054 (2)	0.1991 (3)	0.0352 (9)	
C2	0.0182 (4)	0.3454 (3)	0.1500 (4)	0.0685 (16)	
H2A	0.0942	0.3235	0.1342	0.082*	
C3	−0.1055 (5)	0.3172 (3)	0.1236 (4)	0.0732 (17)	
H3A	−0.1102	0.2761	0.0900	0.088*	
C4	−0.2061 (5)	0.4033 (3)	0.1895 (4)	0.0774 (18)	
H4	−0.2837	0.4244	0.2038	0.093*	
C5	−0.0851 (4)	0.4357 (3)	0.2184 (4)	0.0668 (15)	
H5A	−0.0828	0.4775	0.2505	0.080*	
C6	0.1592 (4)	0.4400 (2)	0.2344 (3)	0.0351 (9)	
C7	0.3474 (4)	0.3869 (2)	0.4092 (2)	0.0368 (9)	
C8	0.2620 (4)	0.3310 (2)	0.4126 (3)	0.0453 (11)	
H8	0.2713	0.2909	0.3786	0.054*	
C9	0.1620 (5)	0.3355 (3)	0.4677 (3)	0.0574 (13)	
H9	0.1031	0.2984	0.4711	0.069*	
C10	0.1508 (5)	0.3954 (3)	0.5172 (3)	0.0559 (13)	
C11	0.2374 (5)	0.4504 (3)	0.5151 (3)	0.0557 (13)	
H11	0.2291	0.4899	0.5504	0.067*	
C12	0.3362 (4)	0.4468 (2)	0.4607 (3)	0.0460 (11)	
H12	0.3953	0.4840	0.4581	0.055*	
C13	0.4719 (4)	0.29175 (19)	0.6327 (2)	0.0301 (8)	
C14	0.5961 (4)	0.3167 (2)	0.6178 (3)	0.0482 (11)	
H14	0.6526	0.2894	0.5877	0.058*	
C15	0.6342 (5)	0.3825 (2)	0.6483 (3)	0.0556 (12)	
H15	0.7183	0.3984	0.6390	0.067*	
C16	0.4389 (4)	0.4011 (2)	0.7026 (3)	0.0424 (10)	
H16	0.3832	0.4305	0.7306	0.051*	
C17	0.3926 (4)	0.3353 (2)	0.6764 (2)	0.0363 (9)	
H17	0.3089	0.3205	0.6881	0.044*	
C18	0.4181 (4)	0.22155 (19)	0.6023 (2)	0.0288 (8)	
C19	0.6340 (4)	0.1167 (2)	0.4150 (2)	0.0346 (9)	
C20	0.6736 (4)	0.1792 (3)	0.3808 (3)	0.0514 (11)	
H20	0.6125	0.2151	0.3669	0.062*	
C21	0.8050 (5)	0.1878 (3)	0.3674 (3)	0.0654 (14)	
H21	0.8330	0.2298	0.3443	0.078*	
C22	0.8945 (5)	0.1344 (3)	0.3881 (3)	0.0606 (14)	
C23	0.8545 (5)	0.0717 (3)	0.4216 (3)	0.0581 (13)	
H23	0.9158	0.0357	0.4350	0.070*	
C24	0.7237 (4)	0.0623 (2)	0.4352 (3)	0.0477 (11)	
H24	0.6957	0.0201	0.4576	0.057*	

### Atomic displacement parameters (Å^2^)

**Table:**

	*U*^11^	*U*^22^	*U*^33^	*U*^12^	*U*^13^	*U*^23^
Br1	0.0866 (5)	0.1511 (8)	0.0966 (5)	0.0053 (5)	0.0490 (4)	−0.0107 (5)
Br2	0.0489 (3)	0.1069 (6)	0.1655 (7)	−0.0228 (3)	0.0441 (4)	−0.0377 (5)
S1	0.0289 (5)	0.0374 (6)	0.0504 (6)	0.0021 (4)	0.0004 (4)	−0.0027 (5)
S2	0.0309 (5)	0.0262 (5)	0.0410 (5)	0.0004 (4)	0.0034 (4)	−0.0016 (4)
O1	0.0373 (16)	0.0419 (19)	0.0602 (18)	0.0023 (13)	0.0028 (14)	−0.0199 (15)
O2	0.0537 (18)	0.0331 (17)	0.0663 (19)	0.0106 (14)	0.0074 (15)	−0.0016 (15)
O3	0.0308 (16)	0.064 (2)	0.072 (2)	−0.0041 (15)	−0.0075 (14)	−0.0053 (17)
O4	0.0277 (14)	0.0306 (16)	0.0552 (17)	−0.0032 (12)	0.0079 (12)	0.0012 (13)
O5	0.0397 (16)	0.0371 (17)	0.0483 (16)	0.0046 (13)	0.0015 (13)	0.0059 (13)
O6	0.0444 (16)	0.0295 (17)	0.0488 (16)	−0.0045 (13)	0.0005 (13)	−0.0060 (13)
N1	0.0282 (19)	0.061 (3)	0.070 (3)	−0.0053 (18)	0.0023 (17)	−0.004 (2)
N2	0.0266 (17)	0.030 (2)	0.045 (2)	−0.0025 (14)	0.0027 (15)	−0.0088 (15)
N3	0.0266 (17)	0.0263 (19)	0.052 (2)	−0.0036 (15)	0.0010 (14)	−0.0082 (17)
N4	0.047 (2)	0.030 (2)	0.062 (2)	−0.0042 (17)	−0.0051 (18)	−0.0024 (18)
N5	0.0257 (16)	0.0244 (19)	0.047 (2)	−0.0017 (14)	0.0003 (14)	−0.0068 (15)
N6	0.0396 (19)	0.0233 (19)	0.0373 (18)	−0.0021 (15)	0.0028 (14)	0.0028 (15)
C1	0.029 (2)	0.038 (2)	0.038 (2)	−0.0023 (17)	0.0013 (16)	−0.0056 (18)
C2	0.019 (2)	0.076 (4)	0.110 (4)	0.002 (2)	0.005 (2)	−0.043 (3)
C3	0.039 (3)	0.070 (4)	0.109 (4)	−0.003 (3)	0.000 (3)	−0.045 (3)
C4	0.030 (3)	0.089 (5)	0.115 (5)	0.005 (3)	0.016 (3)	−0.032 (4)
C5	0.034 (3)	0.067 (4)	0.101 (4)	−0.001 (2)	0.013 (3)	−0.039 (3)
C6	0.033 (2)	0.029 (2)	0.042 (2)	0.0000 (18)	0.0043 (18)	0.0011 (19)
C7	0.036 (2)	0.032 (2)	0.040 (2)	0.0027 (18)	−0.0041 (17)	0.0008 (19)
C8	0.050 (3)	0.035 (3)	0.050 (3)	−0.001 (2)	0.004 (2)	−0.003 (2)
C9	0.054 (3)	0.054 (3)	0.064 (3)	−0.014 (2)	0.007 (2)	0.007 (3)
C10	0.053 (3)	0.068 (4)	0.047 (3)	0.010 (3)	0.009 (2)	0.002 (3)
C11	0.064 (3)	0.056 (3)	0.046 (3)	0.010 (3)	0.002 (2)	−0.009 (2)
C12	0.050 (3)	0.042 (3)	0.044 (2)	0.001 (2)	−0.006 (2)	−0.004 (2)
C13	0.0307 (19)	0.025 (2)	0.034 (2)	−0.0031 (16)	0.0013 (16)	0.0014 (17)
C14	0.036 (2)	0.031 (2)	0.080 (3)	0.0004 (19)	0.017 (2)	−0.007 (2)
C15	0.039 (2)	0.036 (3)	0.091 (4)	−0.010 (2)	0.009 (2)	−0.004 (3)
C16	0.055 (3)	0.032 (3)	0.041 (2)	0.007 (2)	0.005 (2)	−0.0044 (19)
C17	0.038 (2)	0.031 (2)	0.040 (2)	−0.0023 (18)	0.0058 (18)	−0.0013 (18)
C18	0.029 (2)	0.025 (2)	0.031 (2)	−0.0024 (16)	0.0007 (16)	0.0026 (16)
C19	0.034 (2)	0.031 (2)	0.040 (2)	−0.0039 (18)	0.0096 (17)	−0.0066 (18)
C20	0.044 (3)	0.048 (3)	0.063 (3)	−0.003 (2)	0.008 (2)	0.004 (2)
C21	0.059 (3)	0.059 (4)	0.081 (4)	−0.021 (3)	0.019 (3)	−0.004 (3)
C22	0.037 (3)	0.068 (4)	0.079 (3)	−0.008 (3)	0.018 (2)	−0.023 (3)
C23	0.043 (3)	0.052 (3)	0.081 (3)	0.005 (2)	0.011 (2)	−0.017 (3)
C24	0.044 (3)	0.033 (3)	0.068 (3)	−0.001 (2)	0.015 (2)	−0.001 (2)

### Geometric parameters (Å, °)

**Table:**

Br1—C10	1.893 (5)	C4—H4	0.9300
Br2—C22	1.891 (4)	C5—H5A	0.9300
S1—O2	1.426 (3)	C7—C8	1.376 (6)
S1—O3	1.427 (3)	C7—C12	1.392 (6)
S1—N3	1.661 (4)	C8—C9	1.386 (6)
S1—C7	1.758 (4)	C8—H8	0.9300
S2—O5	1.421 (3)	C9—C10	1.375 (7)
S2—O6	1.432 (3)	C9—H9	0.9300
S2—N6	1.654 (3)	C10—C11	1.370 (7)
S2—C19	1.760 (4)	C11—C12	1.369 (6)
O1—C6	1.213 (5)	C11—H11	0.9300
O4—C18	1.216 (4)	C12—H12	0.9300
N1—C3	1.306 (6)	C13—C17	1.375 (5)
N1—C4	1.313 (6)	C13—C14	1.387 (5)
N2—C6	1.343 (5)	C13—C18	1.495 (5)
N2—N3	1.409 (4)	C14—C15	1.375 (6)
N2—H2	0.75 (4)	C14—H14	0.9300
N3—H3	0.77 (4)	C15—H15	0.9300
N4—C15	1.322 (6)	C16—C17	1.379 (5)
N4—C16	1.323 (5)	C16—H16	0.9300
N5—C18	1.348 (5)	C17—H17	0.9300
N5—N6	1.403 (4)	C19—C20	1.375 (6)
N5—H5	0.83 (4)	C19—C24	1.386 (6)
N6—H6	0.83 (4)	C20—C21	1.379 (6)
C1—C5	1.351 (6)	C20—H20	0.9300
C1—C2	1.359 (6)	C21—C22	1.372 (7)
C1—C6	1.513 (5)	C21—H21	0.9300
C2—C3	1.377 (6)	C22—C23	1.376 (7)
C2—H2A	0.9300	C23—C24	1.376 (6)
C3—H3A	0.9300	C23—H23	0.9300
C4—C5	1.393 (7)	C24—H24	0.9300
			
O2—S1—O3	120.34 (19)	C9—C8—H8	120.6
O2—S1—N3	106.87 (17)	C10—C9—C8	119.3 (4)
O3—S1—N3	104.46 (18)	C10—C9—H9	120.3
O2—S1—C7	108.10 (19)	C8—C9—H9	120.3
O3—S1—C7	110.28 (19)	C11—C10—C9	121.9 (4)
N3—S1—C7	105.78 (17)	C11—C10—Br1	118.2 (4)
O5—S2—O6	120.20 (17)	C9—C10—Br1	119.9 (4)
O5—S2—N6	107.03 (17)	C12—C11—C10	119.4 (4)
O6—S2—N6	104.08 (17)	C12—C11—H11	120.3
O5—S2—C19	108.57 (18)	C10—C11—H11	120.3
O6—S2—C19	109.09 (18)	C11—C12—C7	119.3 (4)
N6—S2—C19	107.09 (17)	C11—C12—H12	120.4
C3—N1—C4	116.3 (4)	C7—C12—H12	120.4
C6—N2—N3	120.9 (3)	C17—C13—C14	117.6 (4)
C6—N2—H2	124 (3)	C17—C13—C18	118.3 (3)
N3—N2—H2	115 (3)	C14—C13—C18	124.1 (3)
N2—N3—S1	112.5 (3)	C15—C14—C13	118.7 (4)
N2—N3—H3	114 (3)	C15—C14—H14	120.6
S1—N3—H3	109 (3)	C13—C14—H14	120.6
C15—N4—C16	117.0 (4)	N4—C15—C14	123.9 (4)
C18—N5—N6	118.1 (3)	N4—C15—H15	118.1
C18—N5—H5	125 (3)	C14—C15—H15	118.1
N6—N5—H5	114 (3)	N4—C16—C17	123.5 (4)
N5—N6—S2	113.2 (2)	N4—C16—H16	118.2
N5—N6—H6	111 (3)	C17—C16—H16	118.2
S2—N6—H6	120 (3)	C13—C17—C16	119.2 (4)
C5—C1—C2	117.5 (4)	C13—C17—H17	120.4
C5—C1—C6	118.0 (4)	C16—C17—H17	120.4
C2—C1—C6	124.5 (4)	O4—C18—N5	123.8 (3)
C1—C2—C3	119.7 (4)	O4—C18—C13	121.0 (3)
C1—C2—H2A	120.2	N5—C18—C13	115.2 (3)
C3—C2—H2A	120.2	C20—C19—C24	121.0 (4)
N1—C3—C2	123.8 (5)	C20—C19—S2	120.1 (3)
N1—C3—H3A	118.1	C24—C19—S2	118.9 (3)
C2—C3—H3A	118.1	C19—C20—C21	119.2 (5)
N1—C4—C5	123.6 (4)	C19—C20—H20	120.4
N1—C4—H4	118.2	C21—C20—H20	120.4
C5—C4—H4	118.2	C22—C21—C20	120.1 (5)
C1—C5—C4	119.0 (5)	C22—C21—H21	120.0
C1—C5—H5A	120.5	C20—C21—H21	120.0
C4—C5—H5A	120.5	C21—C22—C23	120.7 (4)
O1—C6—N2	122.8 (4)	C21—C22—Br2	120.8 (4)
O1—C6—C1	121.0 (3)	C23—C22—Br2	118.5 (4)
N2—C6—C1	116.2 (3)	C24—C23—C22	119.8 (5)
C8—C7—C12	121.3 (4)	C24—C23—H23	120.1
C8—C7—S1	120.1 (3)	C22—C23—H23	120.1
C12—C7—S1	118.4 (3)	C23—C24—C19	119.2 (4)
C7—C8—C9	118.8 (4)	C23—C24—H24	120.4
C7—C8—H8	120.6	C19—C24—H24	120.4
			
C6—N2—N3—S1	97.7 (4)	Br1—C10—C11—C12	−180.0 (3)
O2—S1—N3—N2	60.2 (3)	C10—C11—C12—C7	−0.6 (6)
O3—S1—N3—N2	−171.2 (3)	C8—C7—C12—C11	−0.7 (6)
C7—S1—N3—N2	−54.8 (3)	S1—C7—C12—C11	173.8 (3)
C18—N5—N6—S2	103.9 (3)	C17—C13—C14—C15	1.0 (6)
O5—S2—N6—N5	−55.9 (3)	C18—C13—C14—C15	179.4 (4)
O6—S2—N6—N5	175.8 (2)	C16—N4—C15—C14	−0.1 (7)
C19—S2—N6—N5	60.3 (3)	C13—C14—C15—N4	−1.2 (7)
C5—C1—C2—C3	−1.4 (8)	C15—N4—C16—C17	1.7 (6)
C6—C1—C2—C3	177.8 (5)	C14—C13—C17—C16	0.4 (6)
C4—N1—C3—C2	1.2 (9)	C18—C13—C17—C16	−178.1 (3)
C1—C2—C3—N1	−0.1 (10)	N4—C16—C17—C13	−1.8 (6)
C3—N1—C4—C5	−0.8 (9)	N6—N5—C18—O4	3.7 (5)
C2—C1—C5—C4	1.7 (8)	N6—N5—C18—C13	−175.0 (3)
C6—C1—C5—C4	−177.5 (5)	C17—C13—C18—O4	16.8 (5)
N1—C4—C5—C1	−0.7 (9)	C14—C13—C18—O4	−161.5 (4)
N3—N2—C6—O1	4.6 (6)	C17—C13—C18—N5	−164.5 (3)
N3—N2—C6—C1	−174.9 (3)	C14—C13—C18—N5	17.2 (5)
C5—C1—C6—O1	3.2 (6)	O5—S2—C19—C20	10.3 (4)
C2—C1—C6—O1	−176.0 (5)	O6—S2—C19—C20	143.0 (3)
C5—C1—C6—N2	−177.3 (4)	N6—S2—C19—C20	−105.0 (4)
C2—C1—C6—N2	3.5 (6)	O5—S2—C19—C24	−170.3 (3)
O2—S1—C7—C8	−16.3 (4)	O6—S2—C19—C24	−37.6 (4)
O3—S1—C7—C8	−149.7 (3)	N6—S2—C19—C24	74.4 (4)
N3—S1—C7—C8	97.9 (3)	C24—C19—C20—C21	−0.7 (7)
O2—S1—C7—C12	169.1 (3)	S2—C19—C20—C21	178.7 (4)
O3—S1—C7—C12	35.7 (4)	C19—C20—C21—C22	0.0 (7)
N3—S1—C7—C12	−76.7 (3)	C20—C21—C22—C23	0.6 (8)
C12—C7—C8—C9	1.1 (6)	C20—C21—C22—Br2	−178.2 (4)
S1—C7—C8—C9	−173.4 (3)	C21—C22—C23—C24	−0.5 (8)
C7—C8—C9—C10	−0.1 (7)	Br2—C22—C23—C24	178.3 (4)
C8—C9—C10—C11	−1.2 (7)	C22—C23—C24—C19	−0.2 (7)
C8—C9—C10—Br1	−179.7 (3)	C20—C19—C24—C23	0.8 (6)
C9—C10—C11—C12	1.6 (7)	S2—C19—C24—C23	−178.6 (3)

### Hydrogen-bond geometry (Å, °)

**Table:**

*D*—H···*A*	*D*—H	H···*A*	*D*···*A*	*D*—H···*A*
N2—H2···O4^i^	0.76 (4)	2.15 (4)	2.882 (4)	165.09
N3—H3···N4^ii^	0.78 (4)	2.10 (4)	2.868 (5)	168.21
N6—H6···O6^iii^	0.83 (4)	2.26 (4)	2.998 (4)	147.35
N5—H5···N1^iv^	0.83 (4)	2.03 (4)	2.847 (4)	168.32
N3—H3···O1	0.78 (4)	2.49 (4)	2.732 (4)	100 (3)

Symmetry codes: (i) *x*, −*y*+1/2, *z*−1/2; (ii) −*x*+1, −*y*+1, −*z*+1; (iii) −*x*+1, −*y*, −*z*+1; (iv) *x*+1, −*y*+1/2, *z*+1/2.
